# Lamb Wave-Based Structural Damage Detection: A Time Series Approach Using Cointegration

**DOI:** 10.3390/ma16216894

**Published:** 2023-10-27

**Authors:** Phong B. Dao

**Affiliations:** Department of Robotics and Mechatronics, Faculty of Mechanical Engineering and Robotics, AGH University of Krakow, Al. Mickiewicza 30, 30-059 Krakow, Poland; phongdao@agh.edu.pl

**Keywords:** structural health monitoring, damage detection, aluminium plate, Lamb waves, temperature effect, cointegration, time series analysis

## Abstract

Although Lamb waves have found extensive use in structural damage detection, their practical applications remain limited. This limitation primarily arises from the intricate nature of Lamb wave propagation modes and the effect of temperature variations. Therefore, rather than directly inspecting and interpreting Lamb wave responses for insights into the structural health, this study proposes a novel approach, based on a two-step cointegration-based computation procedure, for structural damage evaluation using Lamb wave data represented as time series that exhibit some common trends. The first step involves the composition of Lamb wave series sharing a common upward (or downward) trend of temperature. In the second step, the cointegration analysis is applied for each group of Lamb wave series, which represents a certain condition of damage. So, a cointegration analysis model of Lamb wave series is created for each damage condition. The geometrical and statistical features of Lamb wave series and cointegration residual series are used for detecting and distinguishing damage conditions. These features include the shape, peak-to-peak amplitude, and variance of the series. The validity of this method is confirmed through its application to the Lamb wave data collected from both undamaged and damaged aluminium plates subjected to temperature fluctuations. The proposed approach can find its application not only in Lamb wave-based damage detection, but also in other structural health monitoring (SHM) systems where the data can be arranged in the form of sharing common environmental and/or operational trends.

## 1. Introduction

Structural Health Monitoring (SHM) is a cutting-edge technology that plays a crucial role in ensuring the safety and longevity of various structures, ranging from bridges and buildings to aerospace components and mechanical systems. It involves the integration of advanced sensor technologies and data analytics to continuously assess the condition and performance of these structures in real-time [1]. Damage detection typically comprises three key stages [2]: (1) gathering data from the target structure through periodic measurements of dynamic response; (2) identifying damage-sensitive features from the collected data; and (3) determining the current condition of the structure by conducting statistical analysis on these features. Guided wave-based SHM techniques have obtained much interest for structural damage monitoring and detection in thin-plate structures for many years due to their ability to travel over long distances, detect damages of small sizes, and quickly examine large areas of a structure with few transducers [3,4,5,6,7,8,9,10,11,12,13,14,15]. Lamb waves are generated in a structure by applying electrical voltage to the transducers. The propagation of Lamb waves interacts with damage and can be recorded by either the same transducers (in a pulse-echo setup) or different transducers (in a pitch-catch arrangement) [16]. Damage-induced disruptions in the sensing path scatter the propagating wave, leading to alterations in the characteristics of the current Lamb wave response when compared to the original response obtained from the baseline data collected when the structure was in its undamaged state [3,16]. Lamb waves are known to be sensitive to numerous types of damage including cracks, corrosion, and delamination [4,6].

One of the major challenges and difficulties with Lamb wave-based SHM systems is that the method is highly prone to contamination by changing environmental and operational conditions (EOCs). Especially, variations in temperature have significant impacts on the propagation of Lamb waves, which can consequently impose severe limitations on the efficiency of Lamb wave-based damage detection [5]. Various experimental studies have reported the dominant influence of temperature on Lamb wave propagation. The study in [6] showed that the signal alteration caused by temperature variations is more pronounced than that induced by damage. It was reported in [17] that high temperature causes a considerable impact on the adhesive bond between transducers and structure, which can significantly modify the amplitude and phase of signals. A recent review in [18] presented a survey on the effects of varying EOCs on Lamb waves. The review has also highlighted many effective strategies for compensating and/or eliminating the effects of EOCs in Lamb wave-based SHM systems. Another major challenge associated with Lamb wave-based SHM systems is the intricate nature of Lamb wave propagation, which can be attributed to two fundamental reasons, as discussed in [19,20,21]. Firstly, there exists the possibility for an infinite quantity of distinct modes to concurrently appear and propagate within a structure. Secondly, these modes may exhibit dispersion characteristics and overlap in both time and frequency domains. Many solutions were developed for the separation of overlapping Lamb wave signatures, as presented in [4,19,20,21]. However, the results attained have not met the expectation.

To overcome the above-discussed problems this research has investigated an alternative approach, that is, in place of using Lamb waves directly for damage detection, we first analyse Lamb wave signals using an appropriate statistics-based signal processing technique and then interpret the damage-sensitive features obtained. A similar approach was employed in [22], in which a stationarity-based method was developed for Lamb wave-based structural damage detection. The method is based on the analysis of the stationary statistical characteristics of Lamb waves and its relations with damage. Another potential solution considered in this study is the application of the cointegration theory [23,24] for the analysis of Lamb waves. Cointegration is an established technique originally developed in the field of econometrics and statistics. The cointegration-based approach has been applied to SHM in the last few years as a potential data normalisation tool for the removal of long-term common trends, caused by the effects of variability in EOCs, from the measured data. Some selected applications of cointegration-based approaches for SHM can be found in [25,26,27,28,29,30,31,32,33,34,35,36,37]. Since cointegration can efficiently eliminate the impact of EOCs from the SHM data, one obtains cointegration residuals that maintain their sensitivity to damage while becoming independent of EOCs.

In [28], the authors applied cointegration to analyse the data of modal frequencies acquired from a long-span arch bridge in the presence of EOCs. The results have shown that the cointegration-based method could detect structural damage with different degrees of severity. Monitoring techniques based on cointegration were developed for assessing rolling bearings operating under time-varying operational conditions [29], and for local damage detection in gearboxes [30]. In [31], cointegration analysis was utilized in nonlinear vibro-acoustic wave modulations to compensate for the influence of the load variations associated with the vibration excitation. This method was applied to detect damage in a laminated composite plate and a composite sandwich panel. In [33], the authors used cointegration-based strategies to remove the environmental effects from vibration data collected from a historical building. The work in [34] investigated cointegration for the data collected from a full-scale helicopter tail boom subject to fatigue crack growth. The cointegration-based algorithm successfully mitigated temperature-induced strain variations and demonstrated the capability to detect damage growth. In [35], a frequency adjustment technique for continuous beam bridges was introduced, utilizing cointegration analysis while accounting for the influence of temperature and humidity. In [36], cointegration analysis was employed to mitigate the influence of environmental factors in the experimental data gathered from a cable-stayed bridge. This approach enabled early-stage damage detection in the suspension systems of the bridge. Recently, the work in [37] has applied the cointegration-based technique to the challenging case of a large-scale structure, that is, the steel roof of the G. Meazza stadium. The results confirm that the effects of the environmental and operational variables were suppressed, while the presence of anomalies in the structure remained evident and could be identified clearly. The cointegration technique was also employed in [38,39] for wind turbine health monitoring and anomaly detection. In [40], the authors applied the cointegration analysis to detect structural damage in a wind turbine blade. Cointegration was used in combination with nonstationarity tests for monitoring and detecting faults in nonstationary dynamic industrial processes, as reported in [41]. Recently, the study in [42] proposed a Bayesian multivariate cointegration technique for the detection of damage in the blades of a wind turbine. To gain insight into how the cointegration procedure can eliminate common trends caused by EOCs from the recorded data and how it can be used to detect faults or damage through cointegration residuals, interested readers are directed to the study [43].

It should be mentioned that some previous research [5,25,26] has investigated a combination of the cointegration theory [23,24] and Augmented Dickey–Fuller (ADF) test [44] for structural health assessment using Lamb waves. Nevertheless, all of these previous studies applied cointegration directly on the Lamb waves, leading to a situation where the cointegration residuals obtained were not indicative enough to be used as damage-sensitive features. For that reason, the ADF test had to be employed to extract the damage-sensitive information from the cointegration residuals. The combination of these two data analysis techniques makes the method become more complicated, require more computing tasks, and act less effectively in practical applications. Therefore, this study proposes a novel approach, based on a two-step cointegration-based computation procedure, for structural damage evaluation using Lamb wave data represented as time series that exhibit some common trends. The first step involves the composition of Lamb wave series sharing a common upward (or downward) trend of temperature. In the second step, the cointegration analysis is applied for each group of Lamb wave series which represents a certain condition of damage. The geometrical and statistical features of Lamb wave series and cointegration residual series are used for detecting and distinguishing damage conditions. These features include the shape, peak-to-peak amplitude, and variance of the series. The validity of this method is confirmed through its application to the Lamb wave data collected from both undamaged and damaged aluminium plates subjected to temperature fluctuations. To the best of the author’s knowledge, analysing Lamb waves in the form of time series using cointegration for damage detection has never been previously investigated in the literature. The proposed approach can find its application not only in Lamb wave-based damage detection, but also in other SHM systems where the data can be arranged in the form of sharing common environmental and/or operational trends.

The subsequent sections of the paper are structured as follows. Section 2 briefly introduces the cointegration theory. Section 3 presents the Lamb wave experiments under the effect of temperature variations and explains how to compose Lamb wave series which exhibit a common upward (or downward) trend of temperature. A cointegration-based computation procedure for the analysis of Lamb wave series sharing common trends is then introduced. The results of damage detection using Lamb wave series under the influence of varying temperature trends are presented in Section 4. Finally, some conclusions and future works are given in Section 5.

## 2. A Brief Introduction of Cointegration Theory

In prior research [5,25,43], the fundamental principles of cointegration analysis and related subjects, such as time series stationarity and common trend removal, were comprehensively elaborated. So, this paper does not delve deeply into these concepts. Interested readers are directed to those resources for in-depth explanations of cointegration theory. Here, we provide only a concise introduction of cointegration.

In essence, cointegration is a phenomenon where two or more nonstationary variables exhibit a common long-term pattern, meaning that they do not permanently diverge from each other except for short-term fluctuations. To put it differently, when a set of nonstationary time series variables tend to establish and sustain a stable long-term equilibrium relationship, cointegration analysis can be employed to identify and characterise this relationship. Let $Y_{t}=(y_{1t},y_{2t},\ldots ,y_{nt}{)}^{T}$ denote an (n×1) vector of nonstationary time series. This *n*-dimension vector is said to be linearly cointegrated if there exists a cointegrating vector $\beta =(\beta_{1},\beta_{2},\ldots ,\beta_{n}{)}^{T}$ such that

(1) $$\beta^{T}Y_{t}=\beta_{1}y_{1t}+\beta_{2}y_{2t}+\cdot \cdot \cdot +\beta_{n}y_{nt}$$

forms a linear combination of the nonstationary time series in $Y_{t}$ that is stationary. This linear combination, denoted as $u_{t}=\beta^{T}Y_{t}+c$, where c is a constant value, is termed as a cointegration residual, and it signifies a long-term equilibrium relationship among the cointegrated time series [45]. Nevertheless, it is important to note that the cointegrating vector β is not unique, as it can be multiplied by any scalar k, resulting in the same relationship, as shown below.

(2) $$k\cdot \beta^{T}Y_{t}=(\beta^{*}{)}^{T}Y_{t}$$

A normalized cointegrating vector, $\beta =(1,-\beta_{2},\ldots ,-\beta_{n}{)}^{T}$, can be used to uniquely identify β [45]. With this normalization, the cointegrating relationship in Equation (1) can be rewritten as

(3) $$\beta^{T}Y_{t}=y_{1t}-\beta_{2}y_{2t}-\cdots -\beta_{n}y_{nt}$$

or

(4) $$y_{1t}=\beta_{2}y_{2t}+\beta_{3}y_{3t}+\cdots +\beta_{n}y_{nt}+\beta^{T}Y_{t}$$

The cointegration residual ($u_{t}=\beta^{T}Y_{t}+c$) is simply formed by multiplying n vectors of time series in $Y_{t}$ by $\beta^{T}$. Hence, when using the cointegration method, a critical aspect is to determine appropriate normalized cointegrating vectors, aiming to generate stationary cointegration residuals with the common trends being eliminated. To achieve this, Johansen’s cointegration method [24], a sequential process relying on the maximum likelihood estimation (MLE), is commonly employed. The theory underpinning this approach is intricate and is not expounded in this paper. Those interested in the theoretical details of Johansen’s cointegration method are directed to the original work [24], while a more simplified explanation can be found in [5,43]. In this study, we utilized Johansen’s cointegration procedure by employing the function ‘jcitest’ of the MATLAB Econometrics Toolbox [46] to compute the normalized cointegrating vectors β and c.

## 3. Lamb Wave-Based Structural Damage Detection Based on Cointegration Analysis of Lamb Wave Series

### 3.1. Lamb Wave Experiments under the Effect of Temperature Variations

**Figure 1:**
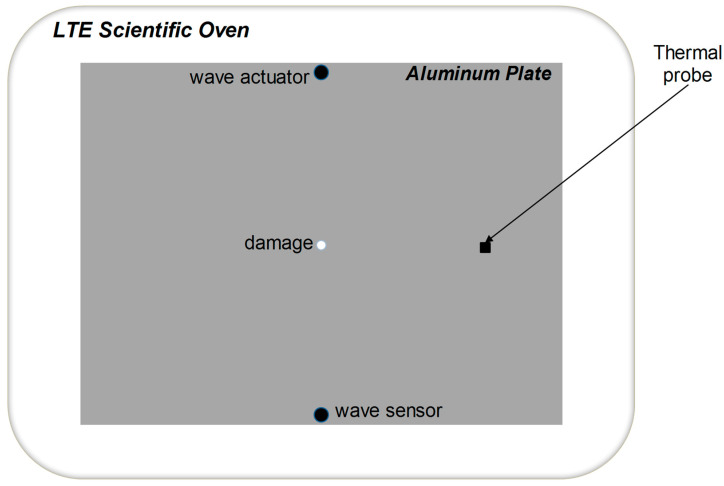
Diagram depicting the experimental arrangement employed for Lamb wave experiments.

The data analysed in this research were obtained from Lamb wave experiments in a pitch-catch arrangement. An aluminium plate specimen with dimensions of 200 × 150 mm and a thickness of 2 mm was used in the experimental process. Two piezoceramic transducers were utilized in a symmetrical arrangement for the purpose of instrumentation. One transducer was responsible for generating Lamb waves, while the other was tasked with capturing the propagation signals. The diameter of both transducers was 10 mm, and their thickness was 1 mm. They were bonded to the specimen’s surface using permanent epoxy adhesive. Lamb waves were generated using a five-cycle 75 kHz cosine burst signal with a maximum peak-to-peak amplitude of 10 V. The excitation signal was enveloped using a half-cosine wave. A diagram depicting the experimental arrangement employed for the Lamb wave experiments is shown in Figure 1, illustrating the specimen’s geometry and the positions of the transducers. To introduce temperature effects into the Lamb wave signals, the specimen was positioned inside a controllable oven during the experimental process. The oven was equipped with a thermal probe for recording the temperature of the plate’s surface. Additional information regarding the experimental configuration can be found in [5,47].

Initially, Lamb wave experiments were conducted on the intact plate. The specimen’s temperature was systematically heated up from 35 °C to 70 °C and then gradually lowered from 70 °C back to 35 °C, with a changing step of 5 °C. Subsequently, two holes, the first one with a 1 mm diameter and then another with a 3 mm diameter, were drilled in the center of the same specimen. The entire test procedure was then repeated to gather experimental data for two different levels of damage severity. Consequently, three distinct damage conditions were examined in this investigation: an undamaged plate, a plate with minor damage severity (1 mm hole), and a plate with substantial damage severity (3 mm hole). For each damage scenario, the Lamb wave response measurements were iterated 50 times at every temperature level, employing a sampling rate of 10 MHz. As a result, we obtained 5000 samples (i.e., sampling points) for each response measurement.

**Figure 2:**
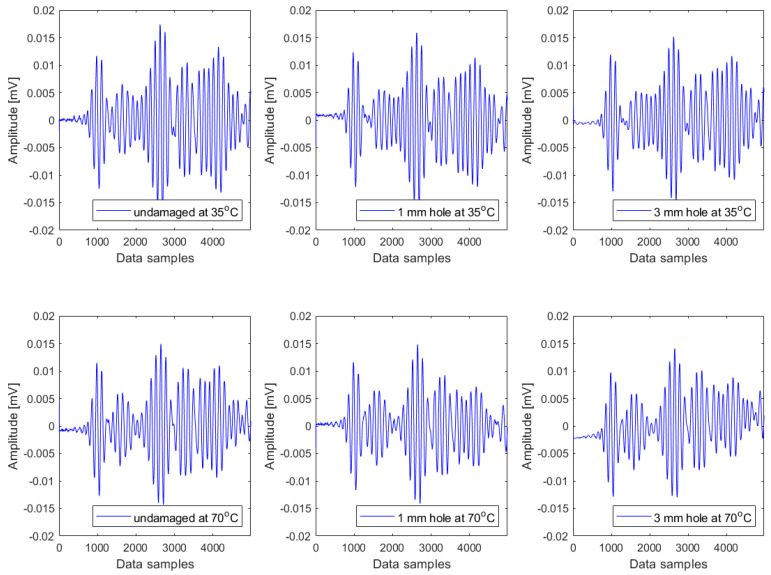
Illustrations of Lamb wave responses representing the intact condition, small damage severity, and large damage severity at 35 °C and 70 °C.

The investigated data were strongly affected by the temperature effect, as reported in [5,47]. As an illustrative example, Lamb wave signals representing three damage conditions measured at two different temperatures (35 °C and 70 °C) are plotted in Figure 2. In all the plots, the signals are characterised by the reflection originating from the structural boundaries and the presence of damage and temperature. As seen in this figure, both damage and temperature have a slight impact on the amplitudes and forms of the Lamb wave responses. These responses encompass various wave packages, including dispersed incident waves, reflected elements, and scattered elements of Lamb wave modes. The alterations in all these responses are attributed to two factors, i.e., structural damage and temperature fluctuations. Figure 2 illustrates that these influences are not readily discernible and pose challenges in their differentiation. Both factors introduce a certain trend into these responses. Nonetheless, it is challenging to recognize those trends in the data. It should be discussed here that the coda wave interferometry (CWI) method, which relies on the cumulative effects of scattered and reflected waves originating from scatterers and structural boundaries, can be used to study the temperature effect in Lamb wave signals. The method has been known for its exceptional sensitivity in detecting small structural damage [48,49].

### 3.2. Composition of Lamb Wave Series Sharing Common Trends

In this study, we introduce a new cointegration-based approach to analyse Lamb wave data. The main idea is that instead of applying cointegration to analyse several Lamb wave responses measured at a specific temperature, as reported in some previous studies [5,25,26], we will use cointegration to analyse Lamb waves as time series variables that exhibit a common trend. The common trend is created by systematically forming the temperature variations in either increasing or decreasing tendency (or direction), as explained below.

The heating phase involves raising the temperature from 35 °C to 70 °C, constituting an upward temperature trend. This trend was constructed in the way that eight Lamb wave responses at 35 °C, 40 °C, …, 65 °C, and 70 °C were sequentially combined in the order of increasing temperatures. This composition procedure resulted in a so-called Lamb wave series, which consists of 40,000 samples. In other words, we created a time series consisting of eight Lamb wave responses arranged in the order of rising temperature. Since the data acquisition was iterated 50 times at every temperature level, these responses can be arbitrarily chosen from any of the repetitions. Nevertheless, for the sake of simplicity, we have opted to select eight Lamb wave responses from the same repetition to create one Lamb wave series. By using this composition procedure, we can create as many Lamb wave series as desired, which are said to exhibit a common upward temperature trend. For cointegration analysis, it is required to have a minimum of two Lamb wave series that exhibit a common trend. Although there is no maximum limit to the number of Lamb wave series that can be analysed using the cointegration algorithm, including too many, Lamb wave series in the analysis is not necessary. In this research, four Lamb wave series have been used to form a cointegration analysis model for each case of damage condition. Regarding the cooling phase from 70 °C to 35 °C, the same composition procedure has been applied to create four Lamb wave series, which exhibit a common downward temperature trend, for each case of damage condition.

**Figure 3:**
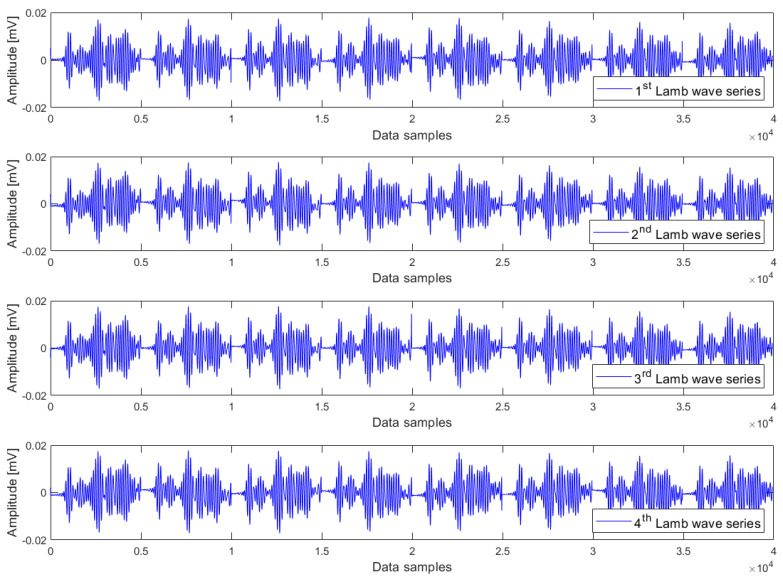
Lamb wave series of the undamaged case exhibiting a common upward trend of temperature.

**Figure 4:**
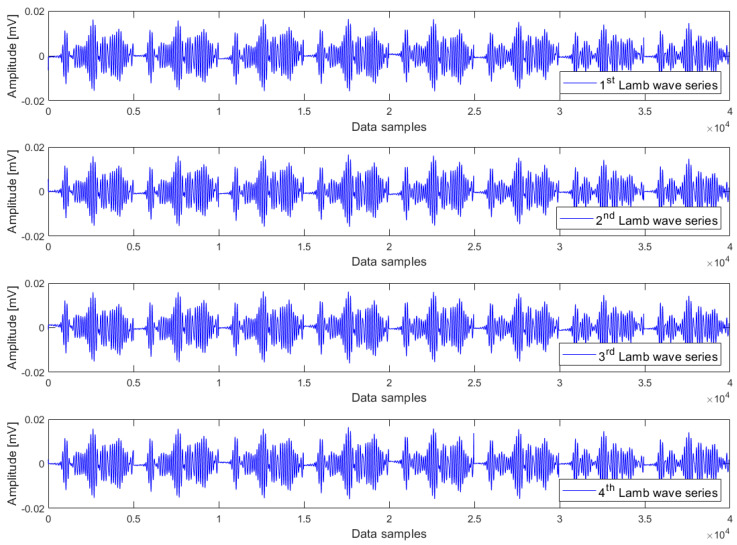
Lamb wave series of the small damage severity (1 mm hole) exhibiting a common upward trend of temperature.

**Figure 5:**
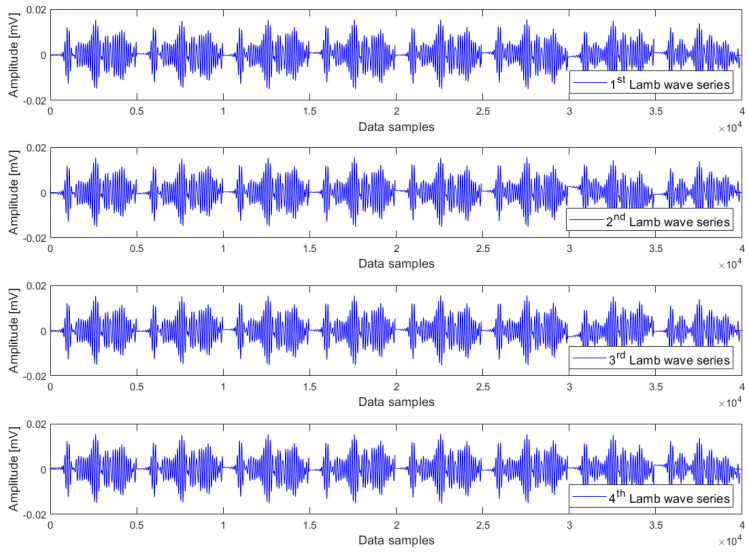
Lamb wave series of the large damage severity (3 mm hole) exhibiting a common upward trend of temperature.

Examples of Lamb wave series for the undamaged case, small damage severity (1 mm hole), and large damage severity (3 mm hole) in the presence of a common upward temperature trend are plotted in Figure 3, Figure 4 and Figure 5 respectively. Obviously, there are no clear differences between Lamb wave series of the same damage condition as well as from different damage conditions. In other words, it is not possible to detect the damage by observing the shape (or form) of these Lamb wave series. The temperature effect exists and causes contamination on the data, but the impact is not observable in these series. The peak-to-peak amplitude and variance of Lamb wave series in Figure 3, Figure 4 and Figure 5 were thus computed for further analysis. The results obtained are the same for all series: the peak-to-peak amplitude ~ [0.032–0.035] mV and the variance ~2.5 × 10^−5^. Hence, it can be inferred that the damage cannot be detected without employing a suitable data analysis method capable of eliminating the temperature-related trend.

### 3.3. Cointegration-Based Computation Procedure for the Analysis of Lamb Wave Series

**Figure 6:**
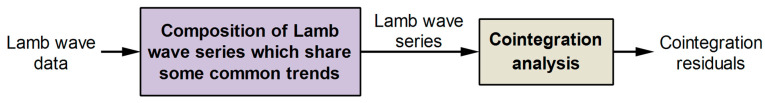
Cointegration-based computation procedure for the analysis of Lamb wave series.

Given that data measurements typically take the form of time-ordered multivariate sequences, it can be understood that a data-based SHM process fundamentally relies on the analysis of time series. We have proposed in this paper a general cointegration-based computation procedure for structural damage evaluation using Lamb wave data represented as time series that have some common trends, as shown in Figure 6.

The first step involves the composition of Lamb wave series which exhibit a common upward (or downward) trend of temperature. This procedure has been explained in Section 3.2. In the second step, cointegration analysis is applied for each group of Lamb wave series which represents a certain condition of damage. In other words, we have created a cointegration analysis model of Lamb wave series for each damage condition. Because the methodology proposed in this study for structural damage detection is based on time series analysis, we have opted to directly use the geometrical and statistical features of Lamb wave series as well as cointegration residual series in order to accurately detect and distinguish damage conditions. These features include the shape, peak-to-peak amplitude, and variance of the time series which can be simply observed or checked visually. When using the shape feature, it is expected that a damage can be detected by means of the direct observation of the shape of a Lamb wave series or a cointegration residual without any computation. In a case where the damage is not clearly noticeable by observing the shape feature directly, then the peak-to-peak amplitude and variance of Lamb wave series or cointegration residuals can be computed. The calculation of these values is straightforward and does not require much computing power and time. This choice of damage-sensitive indicators provides a Lamb wave-based damage detection approach with minimal computation cost.

## 4. Results and Discussion

The cointegration-based computation procedure, described in Section 3.3, was applied to analyse three groups of Lamb wave series which represent three different damage conditions. Two cases of temperature trends, i.e., an upward and a downward, were investigated. The results are presented in the following section.

### 4.1. The Case of Lamb Wave Series Exhibiting a Common Upward Trend of Temperature

Lamb wave series under the influence of an upward temperature trend plotted in Figure 3, Figure 4 and Figure 5 representing the intact case, the small damage severity (1 mm hole), and the large damage severity (3 mm hole), respectively, were cointegrated. So, for each damage condition, we have formed a cointegration analysis model of four Lamb wave series. Johansen’s cointegration procedure was applied by employing the function ‘jcitest’ of the MATLAB Econometrics Toolbox [46] to compute the normalized cointegrating vectors β and the constant value c. As a result, we obtained three cointegration residuals, having the form of $u_{t}=\beta^{T}Y_{t}+c$ for the undamaged case (${res}_{un}$), small damage severity (${res}_{1mm}$), and large damage severity (${res}_{3mm}$). These residuals are given in Equations (5)–(7).

(5) $${res}_{un}=y_{1t}-250.7y_{2t}+39.2y_{3t}-131.2y_{4t}+99.4$$

(6) $${res}_{1mm}=y_{1t}-125.8y_{2t}-108.9y_{3t}-111.2y_{4t}+173.9$$

(7) $${res}_{3mm}=y_{1t}-110.9y_{2t}-199.2y_{3t}-191.1y_{4t}+134.5$$

where $y_{1t},y_{2t},y_{3t},y_{4t}$ corresponds to the Lamb wave series in Figure 3, Figure 4 and Figure 5.

**Figure 7:**
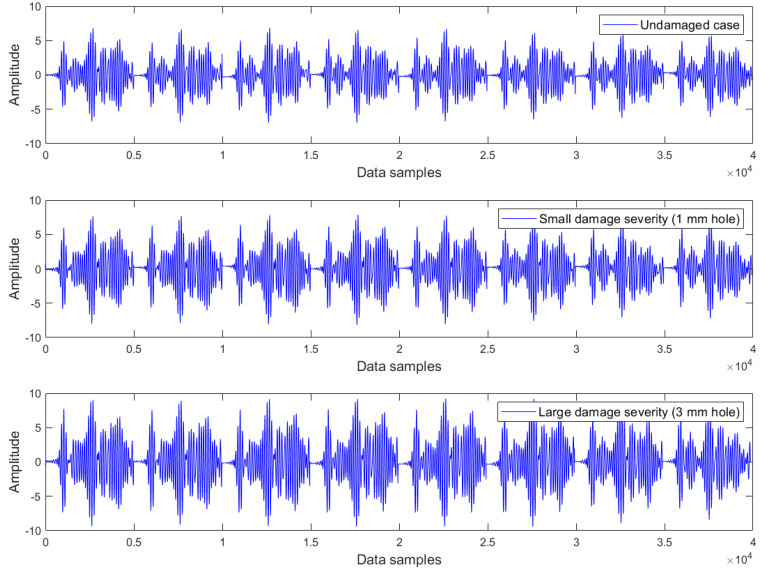
Cointegration residuals obtained for the case of Lamb wave series exhibiting a common upward trend of temperature.

The cointegration analysis results are presented in Figure 7. At first glance, one can notice that all cointegration residuals have a shape (or form) that is similar to Lamb wave series. In addition, the cointegration residual for the undamaged case has the most compact shape, whereas the cointegration residual for the 3 mm hole case exhibits the largest shape. In all cases of the damage conditions investigated, the cointegration analysis removed the effect of temperature, manifested in the form of a common increasing trend of temperature, from the analysed Lamb wave series. At the same time, the effect of damage and its severities still remained and manifested themselves in the cointegration residuals. This is exhibited in the way that the effect of damage and its severities progressively increased the largeness of the cointegration residuals.

Based on these shape differences, we can state that it is possible to detect the damage and its development by observing the shape of cointegration residuals. To quantitatively analyse the results of damage detection, we computed the peak-to-peak amplitude and variance values from the cointegration residuals plotted in Figure 7. The findings, as presented in Table 1, demonstrate that the undamaged state exhibited the lowest peak-to-peak amplitude and variance values, and at the same time, these values were progressively increasing as the severity of damage increased. Hence, this quantitative analysis confirms that not only the existence of damage was detected, but also its severity could be distinguished.

### 4.2. The Case of Lamb Wave Series Exhibiting a Common Downward Trend of Temperature

Three groups of Lamb wave series under the effect of a common downward trend of temperature, representing three different conditions of damage, were cointegrated. So, in this case, we have also obtained three cointegration analysis models of four Lamb wave series. The cointegration residuals, calculated using Johansen’s cointegration procedure, are presented in Equations (8)–(10).

(8) $${res}_{un}=y_{1t}-78.2y_{2t}-210.8y_{3t}-102.6y_{4t}+86.6$$

(9) $${res}_{1mm}=y_{1t}+26.9y_{2t}+223.3y_{3t}+143.9y_{4t}-119.2$$

(10) $${res}_{3mm}=y_{1t}-189.2y_{2t}-111.2y_{3t}-273.6y_{4t}+66.8$$

**Figure 8:**
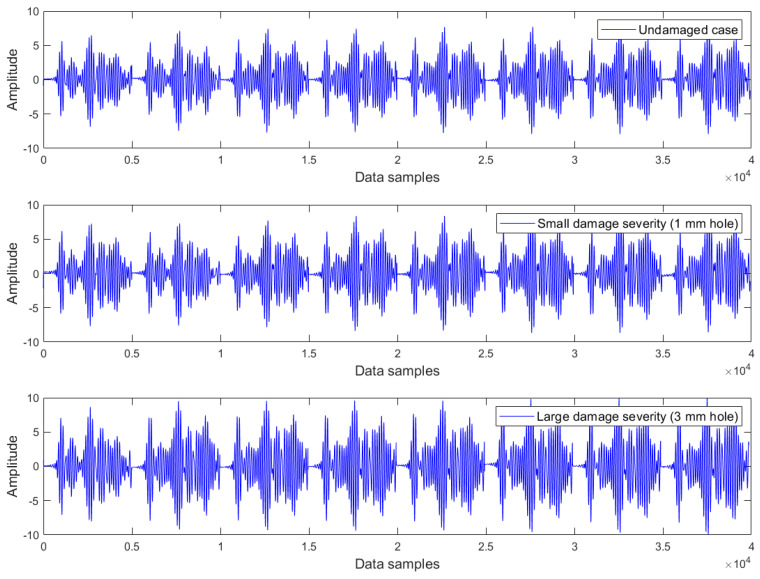
Cointegration residuals obtained for the case of Lamb wave series exhibiting a common downward trend of temperature.

The cointegration analysis results are plotted in Figure 8. Again, the results show that all cointegration residual series exhibit the shape of Lamb waves. Furthermore, the effect of temperature, manifested in the form of a common decreasing trend of temperature, was removed from the analysed Lamb wave series by cointegration. This resulted in three residuals, which were free from the temperature effect, but still captured the impact of damage and its severities. This is illustrated by the fact that the damage severity progressively increased the largeness of the cointegration residuals, as depicted in Figure 8. Consequently, the damage and its severities could be detected by directly using the differences in the shape of these cointegration residuals. The results of damage detection have been quantitatively confirmed by computing the peak-to-peak amplitude and variance values from the cointegration residuals plotted in Figure 8. The results in Table 2 show the same pattern as the case presented in Section 4.1, that is, the undamaged state exhibited the lowest peak-to-peak amplitude and variance values, and these values gradually became greater as the severity of damage increased. The quantitative analysis results confirm that not only the existence of damage was detected, but also its severity was distinguished.

It is noted that all values of the peak-to-peak amplitudes and variances of the cointegration residuals in Table 2 are a bit higher than the relevant ones in Table 1. It is because of the fact that in the case of the cooling stage from 70 °C to 35 °C, the experiments started at the temperature of 70 °C, rather than from 35 °C, as in the case of the heating phase. Also, it is important to mention that the results presented in these two case studies are representative of the entire data sets of the Lamb waves under investigation. The findings obtained for other sets of Lamb wave series closely resemble those presented here.

### 4.3. Discussion

In this study, the proposed two-step cointegration-based computation procedure (shown in Figure 6) has been applied for structural damage evaluation using Lamb wave data presented as time series with a common upward or downward temperature trend. Since the common trend is constituted through a sequential combination of data in either increasing or decreasing temperature order, a prerequisite for this computation procedure is the availability of information about the experimental temperature at which the Lamb wave data were measured. If temperature information is unavailable, the first step of the computation procedure is skipped, and cointegration can be applied directly to analyse Lamb wave responses measured at a specific temperature, as previously reported in [5,25,26]. However, in this case, other data analysis techniques are needed to extract the damage-sensitive information from the cointegration residuals.

## 5. Conclusions

Complex wave propagation phenomena cause numerous challenges and difficulties for damage detection approaches that rely on the direct analysis and interpretation of Lamb waves. Therefore, instead of directly inspecting and interpreting Lamb wave responses for the purpose of structural health assessment, this study has proposed a novel approach, based on a two-step cointegration-based computation procedure, for structural damage evaluation using Lamb wave data formed as time series that share a common upward (or downward) temperature trend. The experimental validation of this method was carried out using Lamb wave data collected from both intact and damaged aluminium plates under the effect of temperature variations. Given the results achieved, we come to the following conclusions:

- The cointegration process could efficiently remove the influence of temperature trends. As a result, the damage can be detected by means of the direct observation of the shapes of the cointegration residuals without any computation.
- It is possible to distinguish the severities of damage by comparing the peak-to-peak amplitude and variance of the cointegration residuals.
- Through the idea of creating the time series of Lamb waves in the form that they exhibit some common trends, we have suggested a new way in applying the cointegration technique for SHM applications.
- The proposed approach is simple and more suitable for practical applications of Lamb wave-based methods because only cointegration is required, without the need for other data analysis techniques.
- Since each Lamb wave series includes a common upward (or downward) trend of temperature which involves multiple levels of temperature, the cointegration process can remove multi-temperature effects, rather than only one temperature when cointegration is applied to directly analyse Lamb wave responses measured at a certain temperature.

This study serves as a feasible exploration, and the results presented in this paper should be regarded as preliminary. Further investigations are thus necessary to validate these findings, encompassing diverse specimen types, more intricate structures, and real-world damage scenarios like fatigue cracks or delamination in metallic structures. Furthermore, future research will explore both the qualitative and quantitative comparisons between the proposed method and other established techniques.

Given that the proposed approach relies on time series cointegration analysis, it has the potential to find applications not only in Lamb wave-based damage detection, but also in other SHM strategies and condition monitoring systems, provided that the experimental data can be arranged in such a way that they share some common environmental and/or operational trends. For instance, this method can be effectively employed for an analysis of the vibration data collected from rotating machinery under varying load, or the natural frequencies of bridges under seasonal or daily temperature changes. It is also expected that potential readers will find the proposed method interesting and attempt to apply it to their own problems.

## Figures and Tables

**Table 1:** Damage detection and discrimination indicated by the calculation results of the peak-to-peak amplitude and variance for the cointegration residuals in Figure 7.

Damage Conditions	Peak-to-Peak Amplitude	Variance
Undamaged case	13.84	4.37
Small damage severity (1 mm hole)	16.02	6.23
Large damage severity (3 mm hole)	18.62	9.25

**Table 2:** Damage detection and discrimination indicated by the calculation results of the peak-to-peak amplitude and variance for the cointegration residuals in Figure 8.

Damage Conditions	Peak-to-Peak Amplitude	Variance
Undamaged case	15.64	6.02
Small damage severity (1 mm hole)	17.32	7.22
Large damage severity (3 mm hole)	20.06	10.23

## Data Availability

No new data were created in this study. Data sharing is not applicable to this article.
